# Genome-wide identification and characterization of multiple C2 domains and transmembrane region proteins in *Gossypium hirsutum*

**DOI:** 10.1186/s12864-020-06842-1

**Published:** 2020-06-29

**Authors:** Pengbo Hao, Hantao Wang, Liang Ma, Aimin Wu, Pengyun Chen, Shuaishuai Cheng, Hengling Wei, Shuxun Yu

**Affiliations:** 1 State Key Laboratory of Cotton Biology, Institute of Cotton Research of CAAS, Anyang, 455000 China; 2 College of Agronomy, Northwest A&F University, Yangling, 712100 China

**Keywords:** *G. hirsutum*, *MCTPs*, N-terminus, C-terminus, Domain architecture, Expression patterns

## Abstract

**Background:**

Multiple C2 domains and transmembrane region proteins (*MCTPs*) may act as transport mediators of other regulators. Although increased number of *MCTPs* in higher plants implies their diverse and specific functions in plant growth and development, only a few plant *MCTPs* have been studied and no study on the *MCTPs* in cotton has been reported.

**Results:**

In this study, we identified 31 *MCTPs* in *G. hirsutum*, which were classified into five subfamilies according to the phylogenetic analysis. GhMCTPs from subfamily V exhibited isoelectric points (pIs) less than 7, whereas GhMCTPs from subfamily I, II, III and IV exhibited pIs more than 7.5, implying their distinct biological functions. In addition, GhMCTPs within subfamily III, IV and V exhibited more diverse physicochemical properties, domain architectures and expression patterns than GhMCTPs within subfamily I and II, suggesting that GhMCTPs within subfamily III, IV and V diverged to perform more diverse and specific functions. Analyses of conserved motifs and pIs indicated that the N-terminus was more divergent than the C-terminus and GhMCTPs’ functional divergence might be mainly contributed by the N-terminus. Furthermore, yeast two-hybrid assay indicated that the N-terminus was responsible to interact with target proteins. Phylogenetic analysis classified multiple N-terminal C2 domains into four subclades, suggesting that these C2 domains performed different molecular functions in mediating the transport of target proteins.

**Conclusions:**

Our systematic characterization of *MCTPs* in *G. hirsutum* will provide helpful information to further research GhMCTPs’ molecular roles in mediating other regulators’ transport to coordinate growth and development of various cotton tissues.

## Background

Intercellular transport of proteins, signaling molecules and carbohydrate is a key process that coordinates the activities of neighboring cells to modulate multicellular organisms’ growth and development [1]. Unlike animal cells, neighboring plant cells are separated by a pair of polysaccharide cell walls [2], which are permeable to small soluble proteins and other solutes, limiting direct contact between adjacent cells [3]. However, Plant have developed plasmodesma (PD) to transport proteins, small RNAs, hormones, and metabolites [4]. One significant feature of the PD is a strand of endoplasmic reticulum (ER) that traverses the pore and is tethered tightly to the plasma membrane (PM) by unidentified spokes [5]. Recent study has demonstrated that multiple C2 domains and transmembrane region proteins (MCTPs) are core PD proteins involved in tethering ER and PM [6].

MCTPs are characterized by three to four C2 domains at the N terminus and one to four transmembrane regions at the C terminus [7]. The C2 domains have been under the enthusiastic research [8–13], because they are the second most ubiquitous lipid binding domain behind the Pleckstrin Homology domain (PH domain) and act as the main sensor of diverse Ca^2+^-mediated cellular processes [14]. The C2 domains were classified into 7 subfamilies [15] and were contained in a large number of proteins that performed distinct physiological functions [16–19]. *MCTP* was first identified in *C. elegans* and function loss of *MCTP* disrupted embryo development [20]. *Drosophila MCTP* was involved in maintaining baseline neurotransmitter release and presynaptic homeostatic plasticity [21]. In mammals, genetic mutations in *MCTPs* might affect the performance of brain and spiral cord, which could lead to bipolar disorder [22, 23]. However, the molecular functions of MCTPs in regulating these processes were still largely unknown, especially the functions of different C2 domains and transmembrane regions contained in MCTPs.

In the plant kingdom, *QKY* and *FTIP1* were the first two reported *MCTPs* in *Arabidopsis* [24, 25]. PD-localized QKY interacted with the receptor-like kinase STRUBBELIG (SUB) to promote cell-to-cell communication and organogenesis [26], while *qky* mutants exhibited twisted gynoecium due to defective cell growth anisotropy and division pattern [27]. ER-localized FTIP1 were the essential intercellular transporter of florigen protein FLOWERING LOCUS T (FT) from companion cells to sieve elements, thereby facilitating FT’s movement from leaves to shoot apical meristem (SAM) and inducing flowering [25]. Thereafter, a genome-wide analysis identified 16 *AtMCTPs,* including *QKY* (*AtMCTP15)* and *FTIP1* (*AtMCTP1*). These AtMCTPs were classified into five clades, which was also supported by phylogenetic analysis of MCTPs in Arabidopsis, rice and several lower plants. Compared with greatly expansion and diversification of MCTPs in seed plants, few MCTPs were found in lycophytes and mosses and were classified into a single clade, representing MCTPs’ early formation in seedless plants. Sixteen AtMCTPs showed diverse expression patterns and subcellular localization, implying MCTPs’ diverse functions in plant development. The authors also demonstrated that three C2 domains contained in FTIP1 might mediate FT’s movement cooperatively [7]. FTIP3/4 (*AtMCTP3/4*) facilitated a key meristem regulator, SHOOTMERISTEMLESS (STM), to recycle to the nucleus to ensure normal maintenance and differentiation of SAM [28]. In orchid, DOFTIP1 interacted with DOFT and promoted flowering [29]. In rice, OsFTIP1 regulated rice flowering time under long days by mediating RFT1’s movement to SAM [30]. Another MCTP of rice, OsFTIP7 contributed to the anther dehiscence through repressing auxin biosynthesis [31]. In maize, ZmCpd33, a homolog of Arabidopsis QKY, promoted symplastic sucrose export from companion cells into sieve elements. The *cpd33* mutants exhibited fewer PD at the companion cell-sieve element interface and excessive carbohydrate accumulation in the leaves [32]. These studies suggest that MCTPs are involved in diverse cellular processes mainly through intercellular or intracellular transport of other regulators.

Upland cotton (*Gossypium hirsutum*) is the most widely cultivated fiber crop for its high productivity and moderate quality of natural textile fiber [33, 34]. As an annual plant with the indeterminate growth habit, upland cotton flowers continuously and periodically from the first flowering to the harvest and subsequently sets spaced bolls on different fruit branches [35]. Both fiber yield and quality are strongly affected by the transport of energy materials and signaling factors among different fruiting sites and vegetative organs. Despite the key roles of MCTPs in the intercellular and intracellular transportation, no *MCTP* was identified in *G. hirsutum* up to now. In this study, we performed the genome-wide identification of GhMCTPs and analyzed their physicochemical properties, phylogenetic relationship with other plants’ MCTPs, gene structures, domain architectures, syntenic relationship and spatiotemporal expression. We also investigated the physicochemical properties of the N-terminal C2 domains and C-terminal transmembrane regions of GhMCTPs, evolutionary divergence of multiple C2 domains and the interaction between GhMCTPs’ C2 domains and GhFT. Our results will be helpful for future characterization of GhMCTPs’ roles in cotton growth and development.

## Results

### Identification, physicochemical properties and chromosomal locations of GhMCTPs

**Fig. 1:**
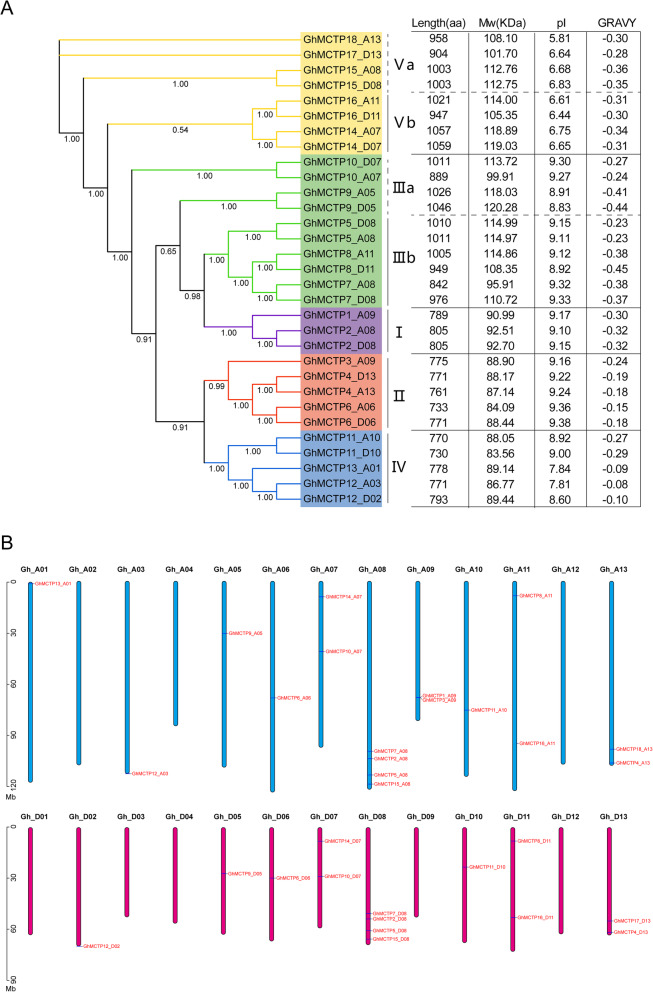
The classification, physiochemical properties and locations on chromosomes of identified GhMCTPs. **a** Thirty one GhMCTPs are classified into five subfamilies according to the phylogenetic tree constructed by MrBayes v3.2.5. Both subfamily III and subfamily V are divided into a and b subclades. The probabilities that support the classified evolutionary subfamilies are marked on the branches of each partition in the tree. The length, Mw, pI and GRAVY are listed in the right table. **b** The locations of *GhMCTPs* on the A and D subgenome are displayed on the blue and red bars, respectively. The lengths of bars represent the lengths of corresponding chromosomes

*AtFTIP1* is one of the well-researched *MCTPs* in *Arabidopsis* [25]. Its protein sequence was used as the query to search against the protein database of *G. hirsutum* for putative GhMCTPs. After confirming the protein domains of the BLAST hits in SMART database, we identified 31 GhMCTPs, each of which contained three to four C2 domains in their N-terminus and one to four transmembrane regions in their C-terminus. The putative GhMCTPs were numbered from 1 to 18 according to their sequence similarity to AtFTIP1 with syntenic GhMCTPs given the same number and a distinct subgenome letter (A or D) (Fig. 5). These GhMCTPs were classified into five subfamilies based on phylogenetic analysis and previous classification of AtMCTPs [7], while both subfamily III and subfamily V were divided into a and b subclades (Fig. 1a). The lengths of GhMCTPs protein sequences ranged from 730 (GhMCTP11_D10) to 1059 (GhMCTP14_D07) amino acids (aa). Correspondingly, GhMCTP11_D10 and GhMCTP14_D07 had the minimum and maximum molecular weight, respectively. The pI and Grand average of hydropathicity (GRAVY) of GhMCTPs ranged from 5.81 to 9.38 and − 0.445 to − 0.075, respectively (Fig. 1a). All the GhMCTPs within the same subfamilies showed distinct GRAVYs, especially GhMCTPs within subfamily III, IV and V. GhMCTPs from subfamily V showed the lowest pIs that were less than 7, indicating their acidic nature and distinct molecular roles from other GhMCTPs. Notably, GhMCTPs within subfamily I and II showed similar pIs, whereas GhMCTPs within subfamily III, IV and V showed different pIs (Fig. 1a), suggesting that GhMCTPs within different subfamilies had experienced different divergences during their evolution.

Thirty one GhMCTPs were unevenly distributed on 18 chromosomes, while the other 8 chromosomes didn’t contain any GhMCTPs. Most of the chromosomes contained 1–2 GhMCTPs, while both A08 and D08 contained 4 GhMCTPs. In addition, A subgenome contained more GhMCTPs than D subgenome (Fig. 1b).

### Phylogenetic analysis of MCTPs in 27 plant species

**Fig. 2:**
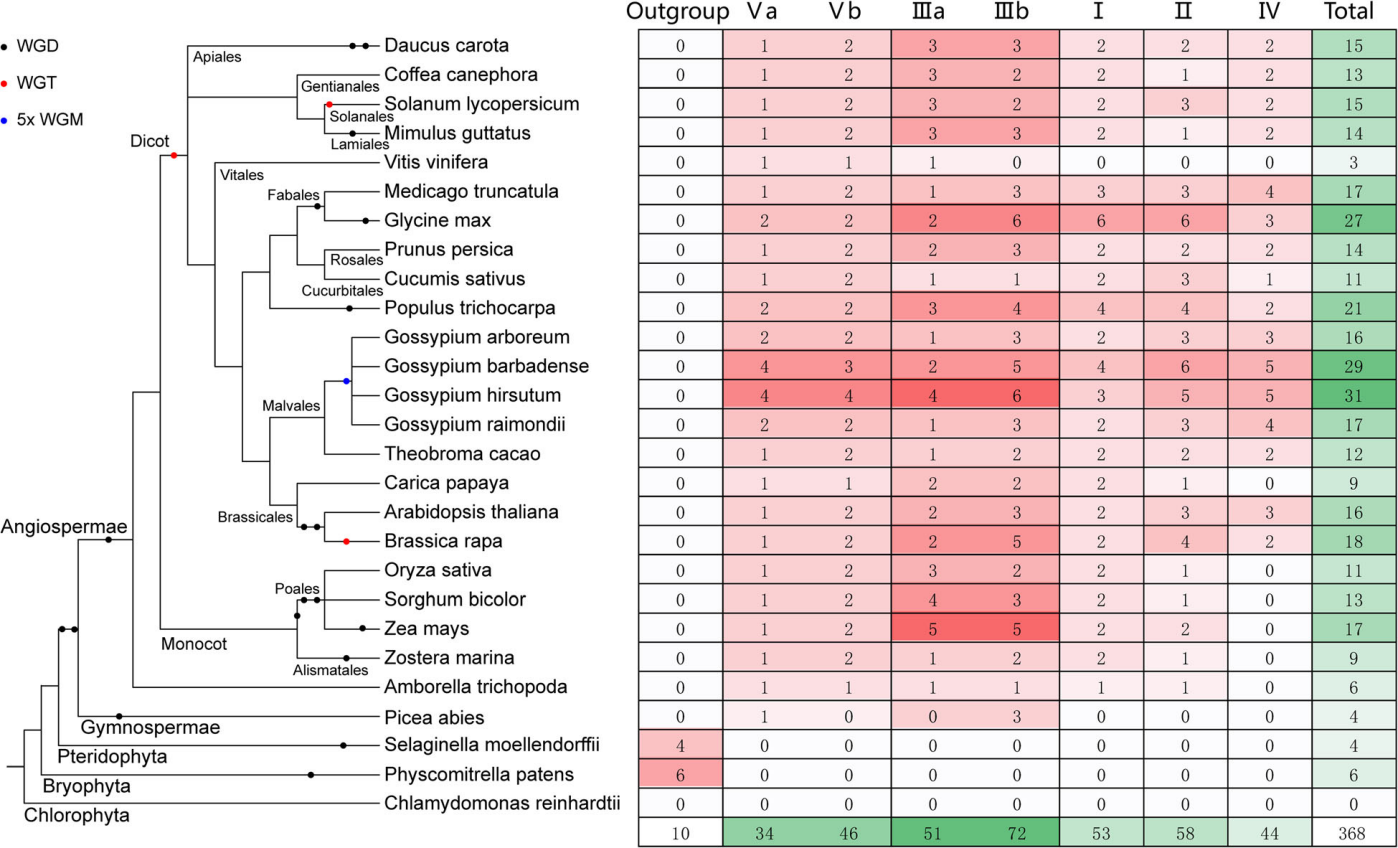
MCTPs’ evolution in 27 plant species. The MCTP numbers from different subfamilies in 27 plant species are listed in the right table corresponding to the left phylogenetic tree of 27 plant species. The red levels illustrate MCTP numbers from different subfamilies in each species. The green levels illustrate total MCTP numbers from all subfamilies in each species and from different subfamilies in all 27 species. The major phyla that 27 plant species belong to and the whole genome duplication events are marked on the corresponding branches of the phylogenetic tree. WGD, WGT and WGM represent whole genome duplication, triplication and multiplication, respectively

To understand the evolutionary relationships among MCTPs in plants, MCTP homologs in *D. carota* (15)*, C. canephora* (13)*, S. lycopersicum* (15)*, M. guttatus* (14)*, V. vinifera* (3)*, M. truncatula* (17)*, G. max* (27)*, P. persica* (14)*, C. sativus* (11)*, P. trichocarpa* (21)*, G. arboreum* (16)*, G. barbadense* (29)*, G. raimondii* (17)*, T. cacao* (12)*, C. papaya* (9)*, A. thaliana* (16)*, B. rapa* (18)*, O. sativa* (11)*, S. bicolor* (13)*, Z. mays* (17)*, Z. marina* (9)*, A. trichopoda* (6)*, P. abies* (4)*, S. moellendorffii* (4), *P. patens* (6)*, C. reinhardtii* (0) were identified with the same method used in GhMCTPs’ identification (Fig. 2). AtMCTPs identified in our study were identical to those identified in the previous study [7]. There was no MCTP identified in chlorophytes (*C. reinhardtii*), suggesting that MCTPs began to form and evolve in terrestrial bryophytes, pteridophytes, gymnosperms and angiosperms (Fig. 2). Different angiosperms had experienced different rounds of whole genome duplications (WGD) [36]. However, MCTP numbers in species that had experienced more WGDs didn’t increase correspondingly compared with MCTP numbers in their close relatives, such as 16 MCTPs in *G. arboreum*, 17 MCTPs in *G. raimondii* compared with 12 MCTPs in *T. cacao* and 18 MCTPs in *B. rapa* compared with 16 MCTPs in *A. thaliana* (Fig. 2)*.* In addition, MCTPs in two AtDt allotetraploids, *G. hirsutum* and *G. barbadense*, were less than the sum of MCTPs in D-genome *G. raimondii* and MCTPs in A-genome *G. arboreum* (Fig. 2). These results suggested that MCTPs experienced gene loss after whole genome duplications. Phylogenetic analysis of 368 MCTPs in 26 plant species classified them into subfamily I-V and one outgroup with 53, 58, 123, 44, 80 and 10 members, respectively. Both subfamily III and subfamily V were divided into a and b subclades. MCTPs within subfamily III, IV and V were more divergent than those within subfamily I and II, which was consistent with different pIs and GRAVYs of GhMCTPs within subfamily III, IV and V (Figs. 1a, 2 and Additional file 1: Figure S1). Six MCTPs in bryophytes (*P. patens*) and four *MCTPs* in pteridophytes (*S. moellendorffii*) were classified into outgroup, which was consistent with the previous classification [7]. It was noteworthy that MCTPs from subfamily V, III and I, II began to evolve in gymnosperms (*P. abies*) and early angiosperms (*A. trichopoda*), respectively, while MCTPs from subfamily IV began to evolve in dicots (Fig. 2). Unexpectedly, there were only 2 MCTPs from subfamily V and 1 MCTP from subfamily III identified in *V. vinifera* (a dicot). These results indicated that the chronological order of MCTPs’ evolution might be outgroup, subfamily V, III, I, II and IV.

### Evolution of intron numbers in *MCTPs*

**Fig. 3:**
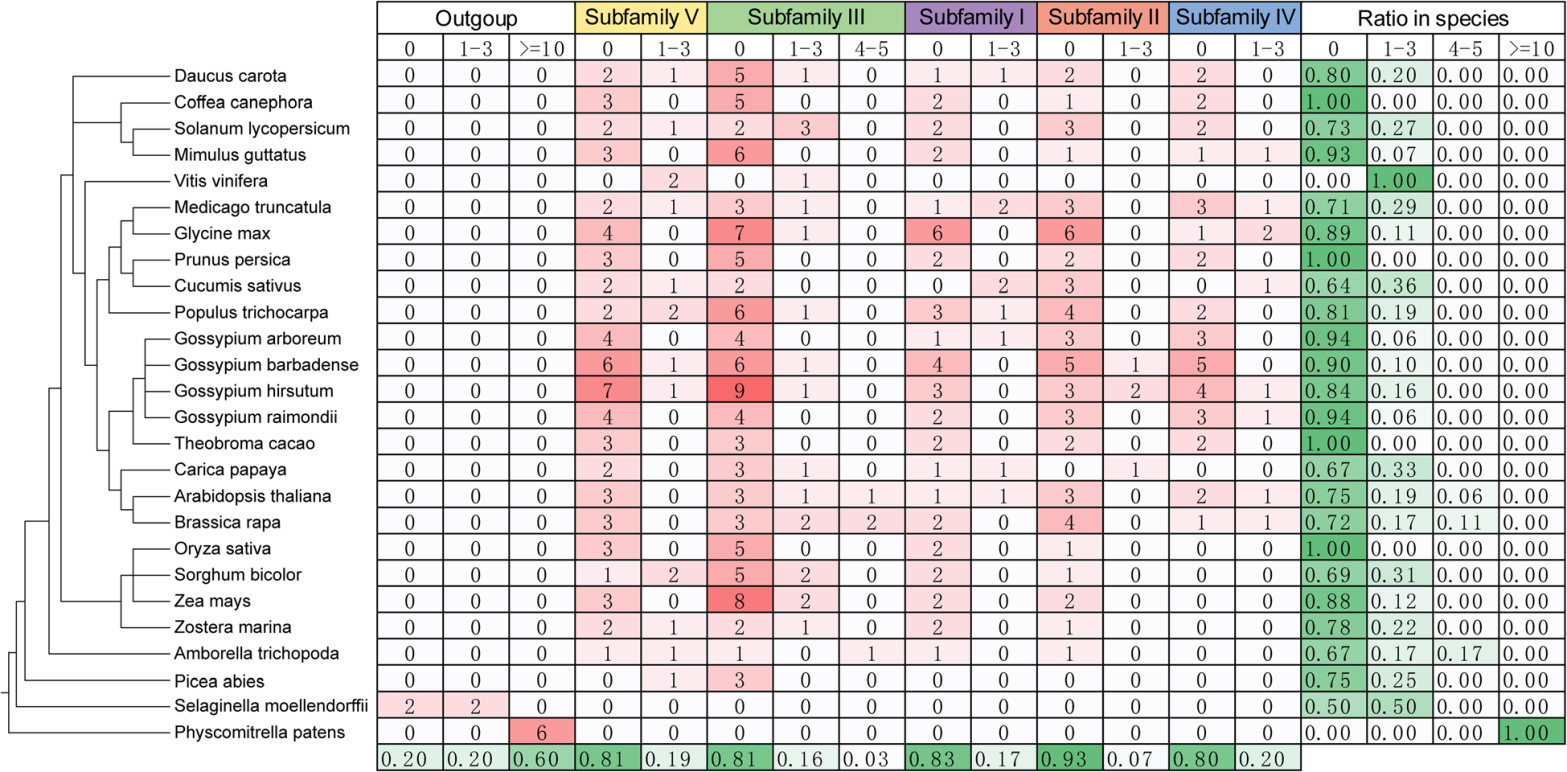
Intron numbers of *MCTPs* from different subfamilies in 26 plant species. The numbers of *MCTP*s containing 0, 1–3, 4–5 and > =10 introns are listed in the right table corresponding to the left phylogenetic tree of 26 plant species. The red levels illustrate the numbers of *MCTPs* containing different numbers of introns from different subfamilies in each species. The green levels illustrate the ratios of *MCTPs* containing different numbers of introns from all subfamilies in each species and from different subfamilies in all 26 species

To better understand the evolution of *MCTPs* in plant species, the intron numbers of 368 *MCTPs* identified in 26 plant species were comparatively analyzed. In bryophytes (*P. patens*), all the MCTPs (6) contained more than 10 introns. In pteridophytes (*S. moellendorffii*), two MCTPs contained 1–3 introns, while another two MCTPs were intronless. In gymnosperms and angiosperms, except that all the MCTPs (3) in *V. vinifera* contained 1–3 introns, ratios of intronless MCTPs in different species diverged significantly, ranging from 0.64 to 1.00 (Fig. 3). These results suggested that MCTPs had experienced drastic intron loss during the speciation of early spermatophytes and the genesis of intron-containing and intronless *MCTPs* were species-specific during the evolution of spermatophytes. Noteworthily, higher ratios of MCTPs from subfamily III (0.19), IV (0.20) and V (0.19) contained introns than MCTPs from subfamily I (0.17) and II (0.07) (Fig. 3), suggesting that not only the protein sequences but also the gene structures of MCTPs within subfamily III, IV and V were more divergent than those of MCTPs within subfamily I and II.

### Domain architectures and conserved motifs of GhMCTPs

**Fig. 4:**
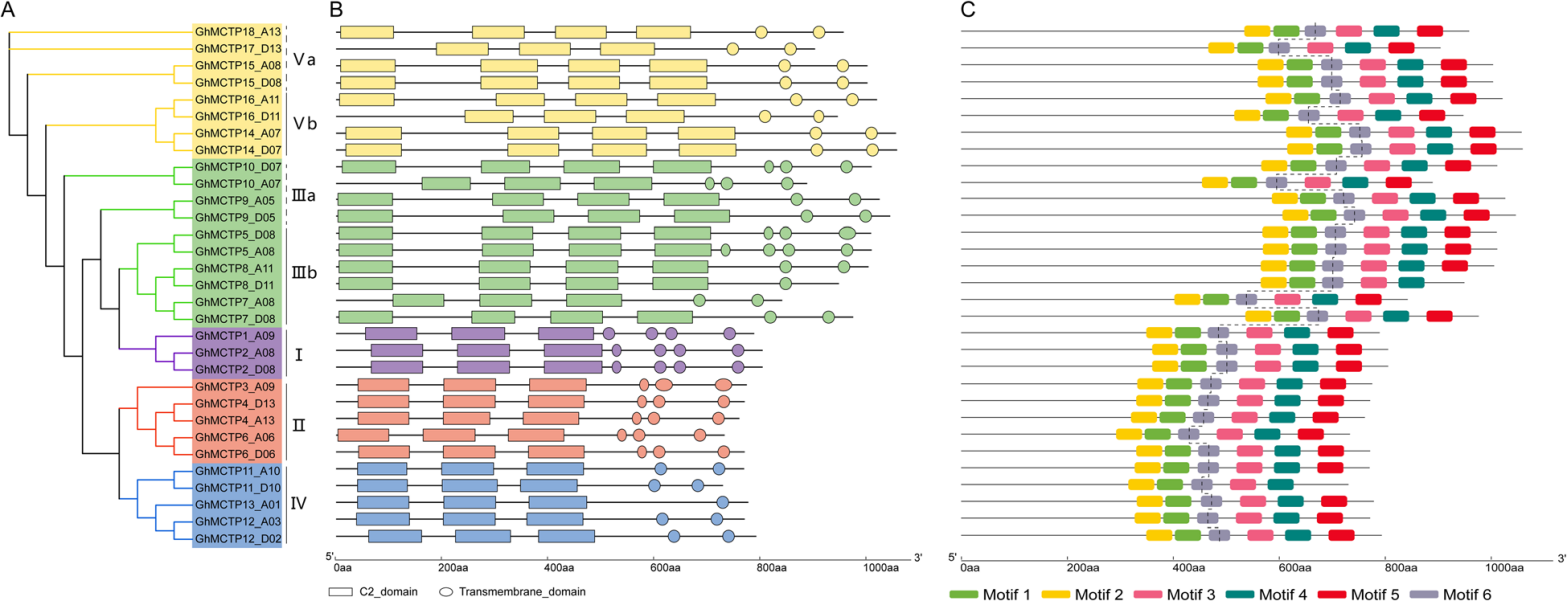
Domain architectures and conserved motifs of GhMCTPs. **a** Phylogenetic tree of GhMCTPs. **b** Domain architectures of GhMCTPs. Rectangles and circles represent C2 domains and transmembrane regions, respectively. **c** Six conserved motifs in GhMCTPs are discovered using MEME. The dotted line represent the border between the N-terminus and C-terminus of GhMCTPs

The conserved domains of GhMCTPs were obtained by searching against the SMART database (Additional file 2: Table S1) and six conserved motifs of GhMCTPs were found using MEME. To further investigate the conservation and diversification of GhMCTPs, the featured domains, 3–4 N-terminal C2 domains and 1–4 C-terminal transmembrane regions, and conserved motifs of GhMCTPs were demonstrated on the phylogenetic tree. All the GhMCTPs from subfamily I, II and IV contained 3 N-terminal C2 domains, whereas most members from subfamily III and V contained 4 N-terminal C2 domains, except GhMCTP7_A08, GhMCTP10_A07, GhMCTP16_D11 and GhMCTP17_D13. Members from subfamily I, II, IV (except GhMCTP13_A01) and V contained 4, 3, 2 and 2 C-terminal transmembrane regions, respectively, whereas members from subfamily III contained 1–4 C-terminal transmembrane regions (Fig. 4b). The transmembrane regions of GhMCTPs were confirmed by TMHMM program (Additional file 3: Figure S2). The different domain architectures of GhMCTPs from different subfamilies hinted their divergent roles in cotton growth and development. However, GhMCTPs within subfamily I and II had similar domain architectures, indicating their functional similarity, while GhMCTPs within subfamily III, IV and V showed relatively divergent domain architectures, which was consistent with their divergent pIs and GRAVYs.

Six conserved motifs were detected in most GhMCTPs, while GhMCTP8_D11 and GhMCTP11_D10 contained five conserved motifs. For most GhMCTPs, motif 1, 2 and partial motif 6 were detected in the end of N-terminus which was the corresponding region of the last C2 domain, while motif 3, 4, 5 and partial motif 6 were detected in the C-terminus. However, no conserved motifs were detected in the most regions of N-terminus (Fig. 4c), suggesting that the last C2 domain and transmembrane regions were more conserved than the other C2 domains, whose divergence might contribute to the structural and functional diversification of GhMCTPs.

### Orthologous *GhMCTPs* between A and D subgenome of *G. hirsutum*

**Fig. 5:**
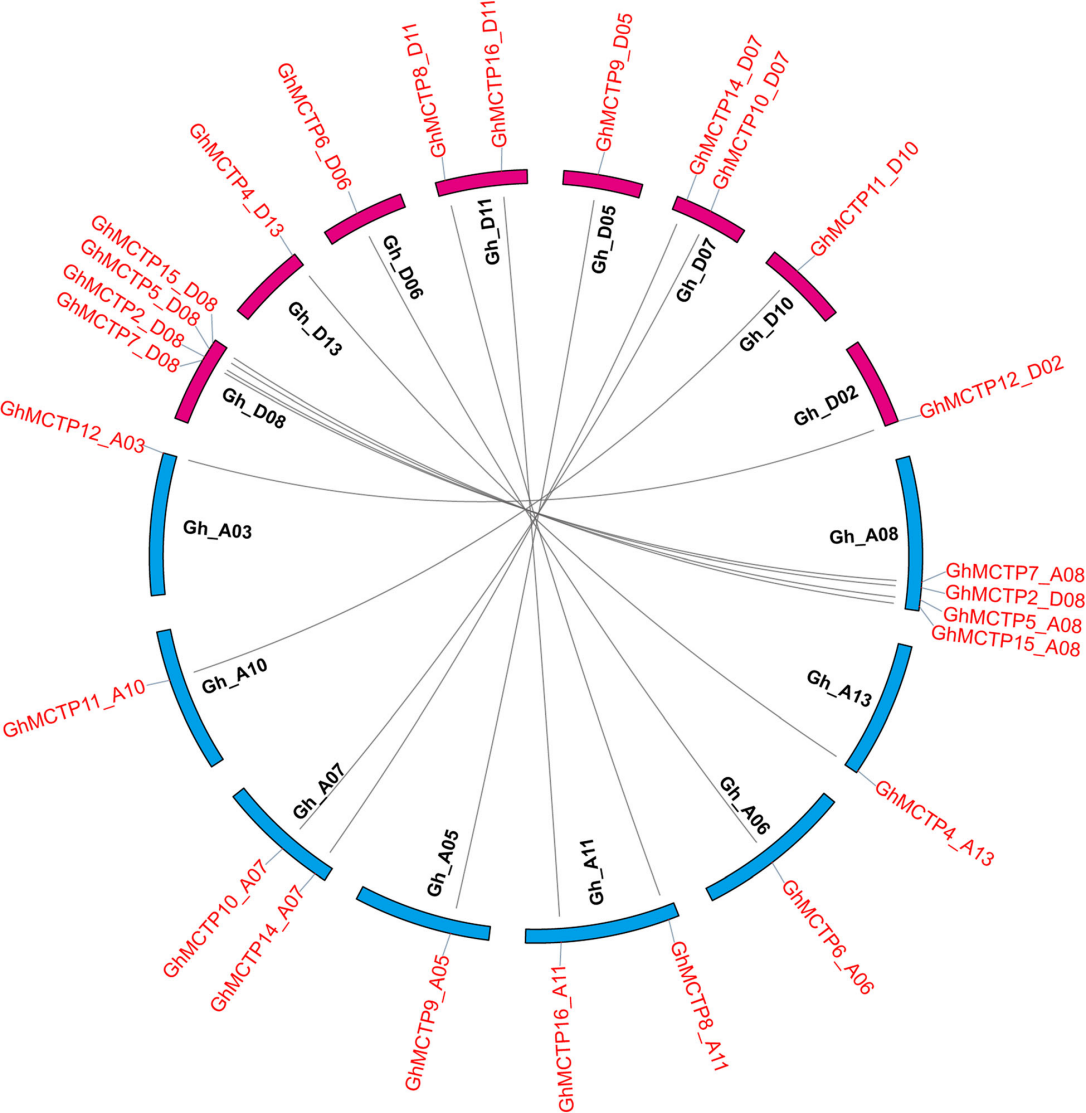
Syntenic *GhMCTPs* between A and D subgenome of *G. hirsutum*. Blue and red bars represent chromosomes from A and D subgenome of *G. hirsutum*, respectively. The grey lines link syntenic *GhMCTPs* detected by MCScanX

To determine whether *GhMCTPs* from A and D subgenome exhibited functional divergence, we identified 13 syntenic pairs of homologous *GhMCTPs* between A and D subgenome of *G. hirsutum* and all these syntenic pairs were located on the similar positions of homologous chromosomes between A and D subgenome, except that *GhMCTP12_A03* and *GhMCTP12_D02* were located on the A03 and D02, respectively (Fig. 5), which might be due to the large reciprocal translocation between A02 and A03 [37]. The synonymous distances (Ks values) between these detected syntenic pairs, partially representing sequence divergence between the two progenitor genomes (A genome and D genome) that formed *G. hirsutum*, ranged from 0.032 to 0.119. According to the Ks values, the divergence times of these syntenic *GhMCTPs* were estimated to be 6.20–22.84 million years ago (MYA), with an average of 12.6 MYA (Table 1). In addition, 13 and 14 syntenic pairs of homologous *MCTPs* found in *G. barbadense, G. raimondii* and *G. arboreum* showed similar ranges of Ks values and divergence times to those in *G. hirsutum* (Additional file 4: Figure S3 and Additional file 5: Table S2), which were wider than the previously estimated divergence time (6.2–7.1 MYA) of A and D progenitor genomes [34]. The Ka/Ks ratios between all the syntenic *MCTPs* were less than 1.0, implying that these syntenic *MCTPs* experienced purifying selection during the divergence of the two progenitor genomes and might perform similar functions.

**Table 1:** Coding sequence divergence between syntenic *GhMCTPs*

Seq_1	Seq_2	Ka	Ks	Ka/Ks	Divegence time (MYA)
*GhMCTP2_A08*	*GhMCTP2_D08*	0.0071	0.0481	0.1479	9.25
*GhMCTP4_A13*	*GhMCTP4_D13*	0.0023	0.0776	0.0294	14.93
*GhMCTP5_A08*	*GhMCTP5_D08*	0.0050	0.0363	0.1374	6.99
*GhMCTP6_A06*	*GhMCTP6_D06*	0.0276	0.0695	0.3964	13.37
*GhMCTP7_A08*	*GhMCTP7_D08*	0.0077	0.0534	0.1445	10.27
*GhMCTP8_A11*	*GhMCTP8_D11*	0.0074	0.0323	0.2285	6.20
*GhMCTP9_A05*	*GhMCTP9_D05*	0.0096	0.0534	0.1788	10.27
*GhMCTP10_A07*	*GhMCTP10_D07*	0.0069	0.0510	0.1354	9.80
*GhMCTP11_A10*	*GhMCTP11_D10*	0.0157	0.0825	0.1907	15.86
*GhMCTP12_A03*	*GhMCTP12_D02*	0.0137	0.0598	0.2287	11.50
*GhMCTP14_A07*	*GhMCTP14_D07*	0.0163	0.0992	0.1643	19.08
*GhMCTP15_A08*	*GhMCTP15_D08*	0.0083	0.0726	0.1143	13.97
*GhMCTP16_A11*	*GhMCTP16_D11*	0.0281	0.1188	0.2368	22.84

### Spatiotemporal expression patterns of *GhMCTPs*

**Fig. 6:**
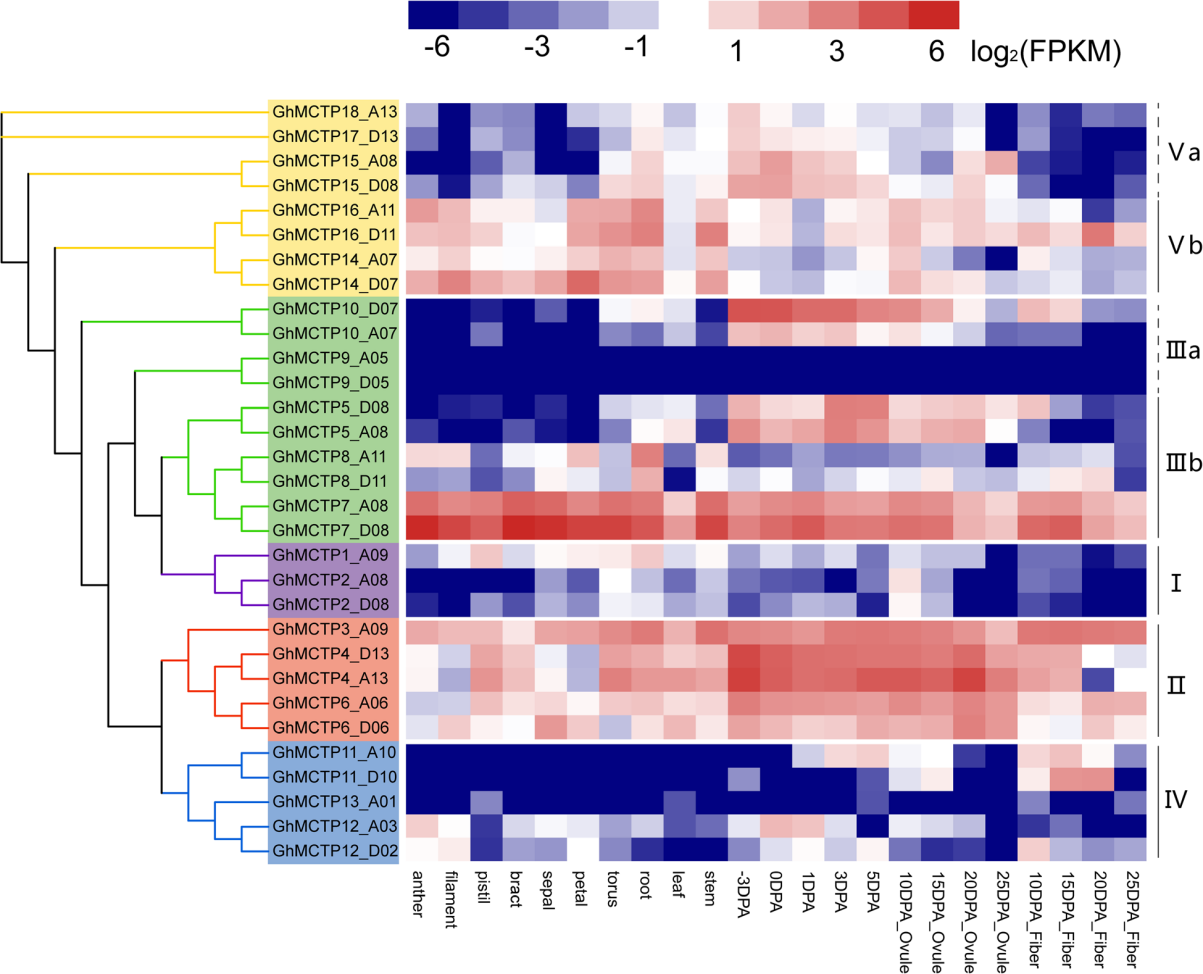
Expression characteristics of *GhMCTPs*. The expression levels of *GhMCTPs* in 23 tissues are displayed on the right of the phylogenetic tree. Differently colored blocks in the scale bar and heatmap represent log2-transformed FPKM values. The investigated tissues are shown on the bottom

The previously published transcriptome datasets of *G. hirsutum* (TM-1) were used to profile the expression of *GhMCTPs* in various tissues, including anther, filament, pistil, bract, sepal, petal, torus, root, leaf, stem, fibers and ovules at different developmental stages [34]. *GhMCTPs* from subfamily II were highly expressed in most tissues, especially in ovules at different developmental stages. *GhMCTP7_A08, GhMCTP7_D08* from subclade IIIb and all the members from subclade Vb also showed high expression levels in most tissues (Fig. 6), suggesting their constitutive roles in the development of various tissues. However, *GhMCTPs* from other subfamilies were only highly expressed in specific tissues. *GhMCTP5_D08, GhMCTP5_A08, GhMCTP10_D07* and *GhMCTP10_A07* from subfamily III were highly expressed in early developmental fibers and ovules at different developmental stages. *GhMCTP11_A10, GhMCTP11_D10* from subfamily IV also showed specific expression in early developmental fibers and ovules at different developmental stages (Fig. 6), suggesting their important roles in ovule and fiber development. These results revealed that *GhMCTPs* from different subfamilies had different expression patterns and might be involved in different biological processes.

### Physicochemically different N-terminus and C-terminus of GhMCTPs

**Fig. 7:**
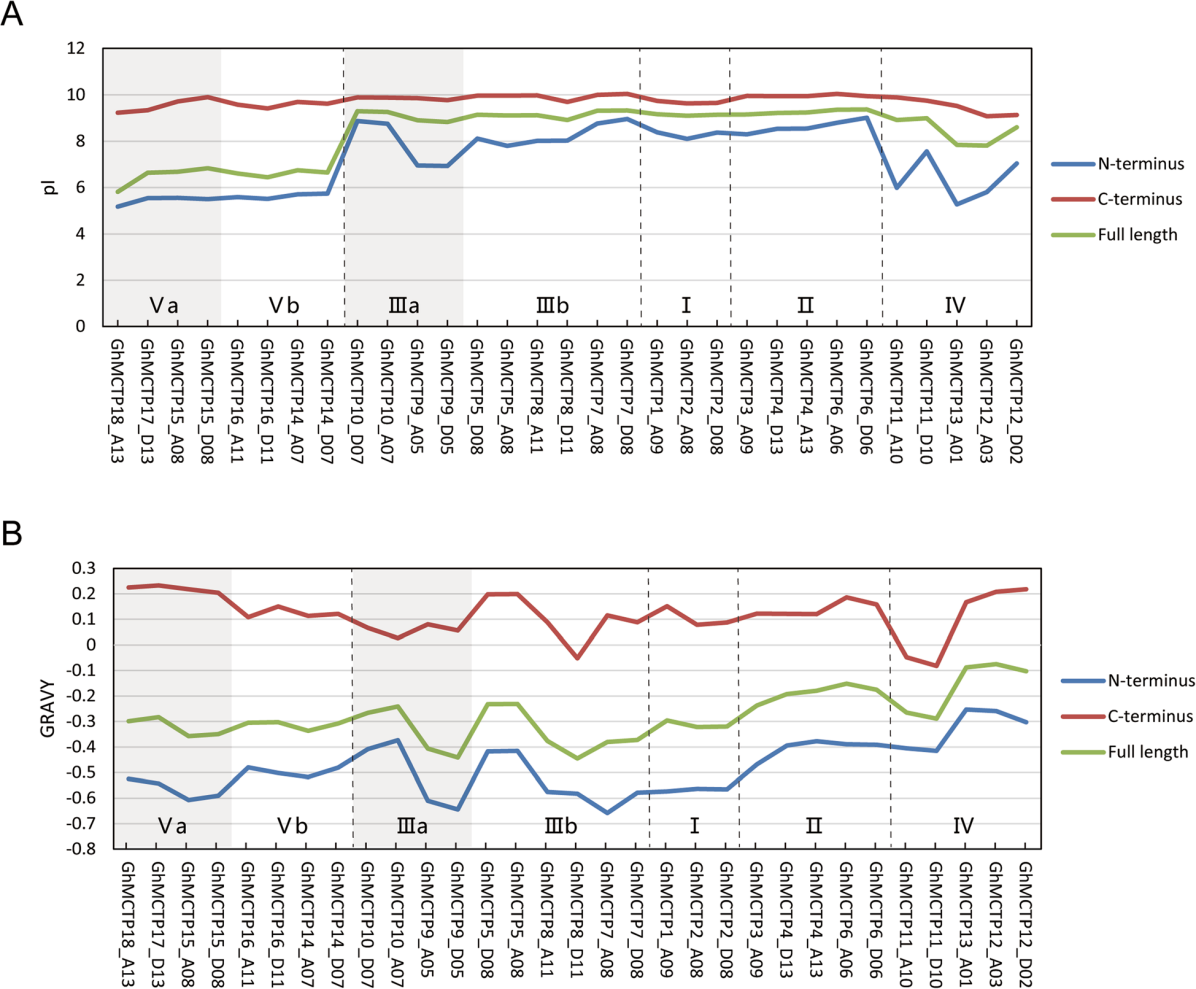
Distinct pIs and GRAVYs between the N-terminus and C-terminus of GhMCTPs. Thirty one GhMCTPs on the X-axis are arranged according to their positions in the phylogenetic tree. Five subfamilies are separated by the dotted lines. **a** The pIs of the N-terminus, C-terminus and full length of 31 GhMCTPs. **b** The GRAVYs of the N-terminus, C-terminus and full length of 31 GhMCTPs

Since the N-terminus and C-terminus of GhMCTPs contained structurally and functionally different domains, which might be reflected by their physicochemical properties, we further analyzed the pIs and GRAVYs of the N-terminus and C-terminus of GhMCTPs (Additional file 6: Table S3). Both pIs and GRAVYs of full-length GhMCTPs were between those of N-terminus and C-terminus, and the C-terminus possessed higher pIs and GRAVYs than the N-terminus. Interestingly, the pIs of the C-terminus were almost invariable among all the GhMCTPs, while the pIs of the N-terminus varied significantly among GhMCTPs from different subfamilies and GhMCTPs within subfamily III and IV (Fig. 7), suggesting that the N-terminus was more variable than the C-terminus and might be the main source of functional divergence of GhMCTPs. However, both the N-terminus and the C-terminus showed significantly different GRAVYs among GhMCTPs within the same subfamilies (Fig. 7).

### Evolutionary divergence of multiple C2 domains in the N-terminus of GhMCTPs

**Fig. 8:**
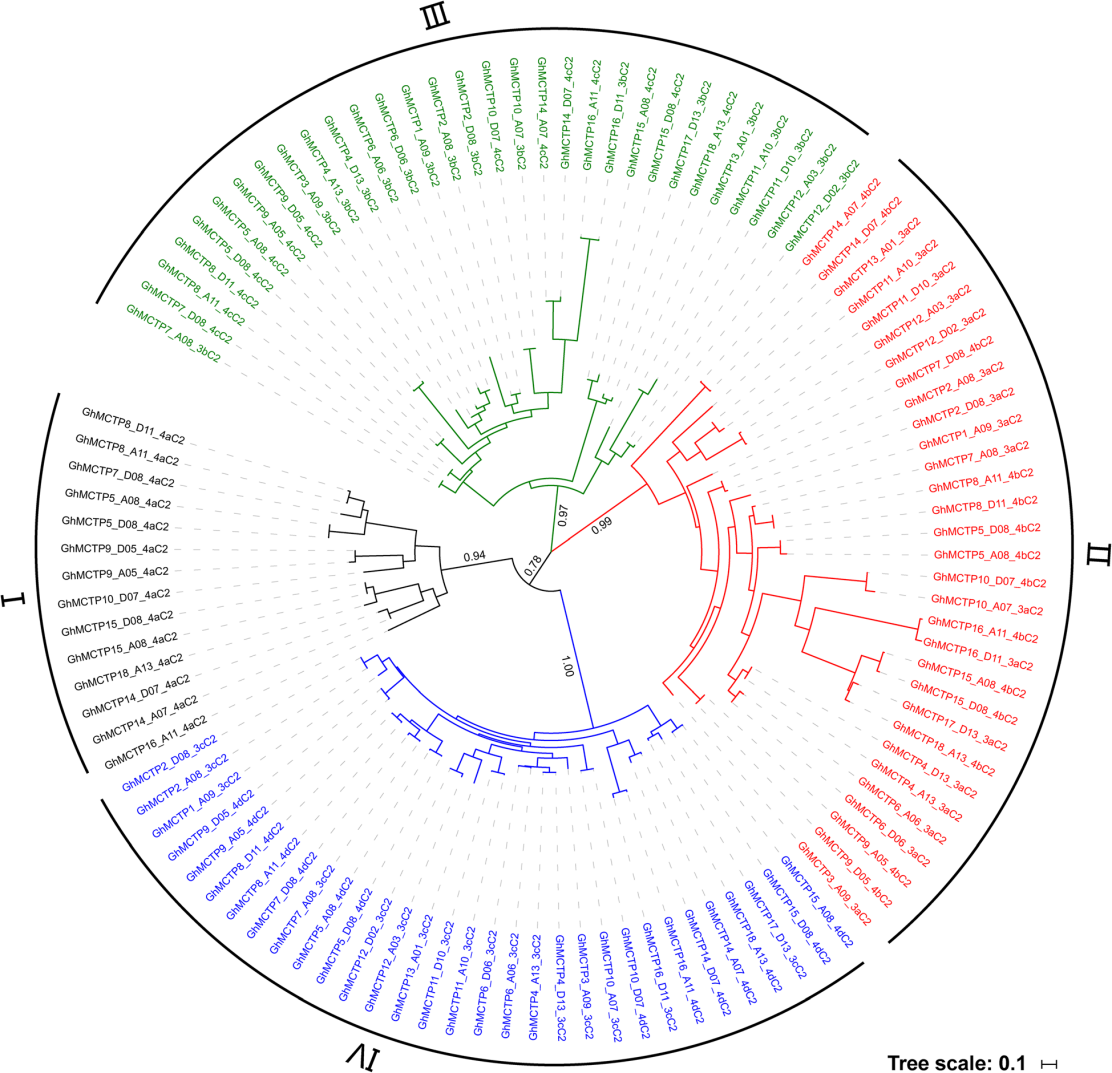
Phylogenetic tree of 107 C2 domains in 31 GhMCTPs. 4aC2, 4bC2, 4cC2, 4dC2 in 4-C2-containing GhMCTPs and 3aC2, 3bC2, 3cC2 in 3-C2-containing GhMCTPs are classified into 4 subclades according to the phylogenetic tree constructed by MrBayes v3.2.5. The probabilities that support the classified evolutionary subfamilies are marked on the branches of each partition in the tree. The tree scale bar represents 0.1 substitutions per amino acid

Since 3–4 C2 domains were contained in the N-terminus of GhMCTPs and showed great difference in protein sequences and physicochemical properties among GhMCTPs, we queried whether the 3–4 C2 domains contained in each of the GhMCTPs had different evolutionary histories or molecular roles and which C2 domain was more divergent among GhMCTPs than the other C2 domains. Four C2 domains of 4-C2-containing GhMCTPs and three C2 domains of 3-C2-containing GhMCTPs were designated as 4aC2, 4bC2, 4cC2, 4dC2 and 3aC2, 3bC2, 3cC2, respectively. The protein sequences of 107 C2 domains contained in 31 GhMCTPs (Additional file 2: Table S1) were used to construct the phylogenetic tree, which classified these C2 domains into subclade I-IV. Consistent with the multiple sequence alignment of the full-length GhMCTPs, in which the 4bC2, 4cC2 and 4dC2 of 4-C2-containing GhMCTPs were aligned with the 3aC2, 3bC2, 3cC2 of 3-C2-containing GhMCTPs, respectively (Additional file 7: Figure S4), the corresponding C2 domains of 4-C2-containing and 3-C2-containing GhMCTPs were classified into the same subclades. In addition, the C2 domains within subclade II and III exhibited larger sequence divergence than those within subclade I and IV (Fig. 8). These results suggested that the 3–4 C2 domains contained in the GhMCTPs began to diverge before the formation of GhMCTPs probably through module exchange and fulfilled different functions in the multidomain GhMCTPs. Moreover, the more divergent 4bC2, 3aC2 and 4cC2, 3bC2 within subclade II and III might be the main source of GhMCTPs’ functional diversification.

### The N-terminus of GhMCTP2_A08 and GhMCTP3_A09 interacted with GhFT

**Fig. 9:**
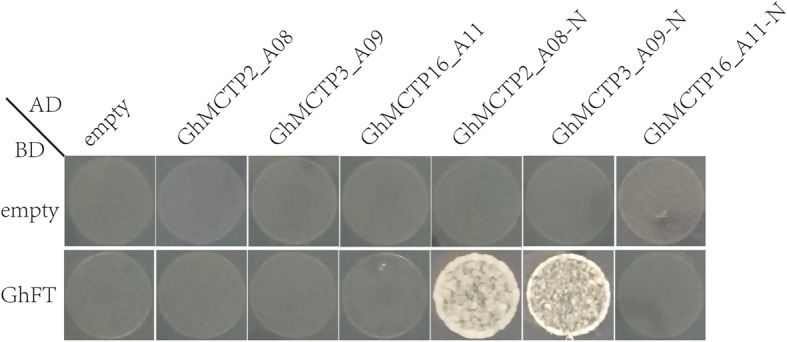
Yeast two-hybrid assay of interaction between GhFT and three GhMCTPs. Yeast cells are co-transformed with recombinant pGADT7 and pGBKT7 vectors and grown on the SD-Trp/−Leu/−His/−Ade/ medium with 10 mM 3-AT (3-amino-1,2,4 -triazole)

The most widely researched MCTPs were FTIP1s [7, 25, 29, 30], which interacted with FTs to mediate their transport from leaves to SAM. We chose three GhMCTPs with different evolutionary distances, including GhMCTP2_A08 (subfamily I), GhMCTP3_A09 (subfamily II) and GhMCTP16_A11 (subfamily V), to detect their interactions with GhFT via yeast two-hybrid assay. All the three full-length GhMCTPs couldn’t interact with GhFT, whereas the N-terminus of GhMCTP2_A08, GhMCTP3_A09 and GhMCTP16_A11 showed strong, weak, and no interaction with GhFT, respectively (Fig. 9). This was consistent with the interaction between FT and N-terminal C2 domains of FTIP1 in *Arabidopsis*, rice and orchid [7, 25, 29, 30]. The transmembrane regions in the C-terminus might hinder GhFT’s interaction with GhMCTP2_A08 and GhMCTP3_A09 in yeast cells. The results suggested that the N-terminal C2 domains of GhMCTPs played key roles in transporting other regulators by direct interaction.

## Discussion

### Sequence characterization of GhMCTPs

Multiple C2 domains and transmembrane region proteins (MCTPs) contain three to four C2 domains in the N terminus and one to four transmembrane regions in the C terminus. MCTPs are evolutionary conserved proteins and have been identified in both animals and plants [7, 21, 24, 25, 27–32, 38]. Compared with animals and lower plants, higher plants contain significantly increased number of MCTPs, implying more diverse and specific functions of MCTPs in plant growth and development [7]. In this study, we identified 31 MCTPs in *G. hirsutum* and classified them into 5 subfamilies (Fig. 1). The distinct physicochemical properties of GhMCTPs suggested that GhMCTPs played diverse roles in regulating cotton growth and development. Especially, all the GhMCTPs from subfamily V exhibited pIs less than 7.5 (Figs. 1a and 7a), the PH of most cell interior compartments [39], suggesting that GhMCTPs from subfamily V and GhMCTPs from other subfamilies were charged oppositely and played different molecular functions in their respective suitable environments.

Phylogenetic analysis of MCTPs in 26 plant species classified these MCTPs into five subfamilies and one outgroup (Fig. 2 and Additional file 1: Figure S1). MCTPs from subfamily V and III, subfamily I and II, subfamily IV successively formed and evolved in gymnosperms, early angiosperms, dicots, respectively (Fig. 2). MCTPs within the ancient subfamily V and III were more divergent and divided into a and b subclades. However, MCTPs from the most recent subfamily IV were also more divergent than MCTPs within another two recent subfamily I and II (Additional file 1: Figure S1), suggesting that MCTPs within subfamily IV had experienced more rapid divergence. These results were consistent with different pIs, GRAVYs and domain architectures of GhMCTPs within subfamily III, IV and V (Figs. 1a, 7 and 4b). The diverse protein sequences, physiochemical properties and domain architectures of GhMCTPs within subfamily III, IV and V indicated these GhMCTPs were involved in more diverse and specific growth and development processes of cotton.

*G. hirsutum* and *G. barbadense* were two allotetraploids that formed ~ 1–2 MYA by the hybridization of two diploids (*G. arboreum* and *G. raimondii*) and the following chromosome doubling [40]. Previous study reported that 369 genes were lost in *G. hirsutum* [37]. The less MCTPs in *G. hirsutum* (31) and *G. barbadense* (29) than the sum (33) of MCTPs in *G. arboreum* and *G. raimondii* suggested that gene losses occurred in both *G. hirsutum* and *G. barbadense* (Fig. 2). The divergence time of A and D progenitor genomes was estimated to be 6.2–7.1 MYA by computing peak Ks values of genome-wide orthologous genes among the four *Gossypium* species [34]. The wider range (6.20–22.84 MYA) of roughly estimated divergence times of syntenic *GhMCTPs* between A and D subgenome (Table 1) might be resulted from varied Ks values among different genes [41] and oversimplified using of general divergence rate for plant nuclear genes in the formula: t = Ks/2r (r = 2.6 × 10^− 9^).

### Diverse expression patterns of *GhMCTPs*

The significantly increased number of MCTPs in higher plants may meet the requirement for more diverse and specific functions of MCTPs in regulating various cellular processes in plants [7]. Functional analyses of MCTPs in various plant species demonstrated that MCTPs were involved in promoting flowering, regulating shoot meristem development, controlling anther dehiscence, regulating cell growth anisotropy and exporting sucrose into sieve elements [7, 24, 25, 27–32]. GUS and GFP reporter assays of 16 AtMCTPs revealed that even MCTPs with close evolutionary relationship might be expressed in different tissues and some MCTPs might perform redundant or additive functions in certain tissues [7]. Our expression analysis showed that *GhMCTPs* from different subfamilies and within the same subfamilies exhibited different spatio-temporal expression patterns, especially *GhMCTPs* within subfamily III, IV and V (Fig. 6), suggesting that *GhMCTPs* played diverse roles in the development of various cotton tissues. *GhMCTP1_A09, GhMCTP2_A08* and *GhMCTP2_D08* within subfamily I exhibited low expression in all the investigated tissues, which might be the result of these *GhMCTPs’* expression at specific locations of these tissues, as *AtMCTP1* and *AtMCTP2’s* expression in the vascular tissues of leaves and roots [7]. Conversely, *GhMCTPs* within subfamily II were highly expressed in most of the investigated tissues, suggesting that these *GhMCTPs* are required to maintain basic cellular processes. The more diverse expression patterns, physicochemical properties and domain architectures of GhMCTPs within subfamily III, IV and V (Figs. 6, 7 and 4b) indicated that GhMCTPs within subfamily III, IV and V evolved to perform more diverse and specific functions than GhMCTPs within subfamily I and II.

### Characterization of multiple C2 domains and transmembrane regions in GhMCTPs

Both C2 domain and transmembrane region are able to target their host proteins to specific organelle membranes, with C2 domain binding to membrane phospholipid mainly in a Ca^2+^-dependent manner [9, 11] and transmembrane region traversing phospholipid bilayer. A recent study on *Arabidopsis* plasmodesmal proteome revealed that MCTPs acted as ER-PM membrane tethers, with C2 domains docking to the PM and transmembrane region inserting into the ER. The distinct physicochemical properties between N-terminal C2 domains and C-terminal transmembrane regions of GhMCTPs (Fig. 7) implied their distinct molecular roles in the interaction with membrane. Compared with the N-terminus, the C-terminus exhibited almost invariable pIs among all the GhMCTPs. In addition, conserved motifs were detected in the C-terminus but not in most regions of the N-terminus (Fig. 4c). These results indicated that the C-terminal transmembrane regions were more conserved than the N-terminal C2 domains. However, GRAVYs of both the N-terminus and the C-terminus exhibited significant variation among GhMCTPs (Fig. 7), which might contribute to their different binding activities to various membranes whose compositions and physical properties could be very different [42].

MCTPs mediate intercellular and intracellular transport of other regulators through the N-terminal C2 domains’ interaction with these regulators [7, 25, 28–31]. Yeast-two hybrid assay showed that the last C2 domain next to transmembrane regions directly interacted with target proteins [7, 30, 31]. The N-terminal C2 domains of GhMCTP2_A08, GhMCTP3_A09 and GhMCTP16_A11 exhibited strong, weak and no interaction with GhFT (Fig. 9). GhMCTP2_A08, AtFTIP1 (AtMCTP1) and OsFTIP1 (Os06g41090.1) were from the same subfamily (I) and had similar N-terminal C2 domain architectures (Additional file 1: Figure S1 and Fig. 4b) [7, 30]. The interaction between AtFTIP1, OsFTIP1, GhMCTP2_A08 and AtFT, OsFT, GhFT, respectively, implied that the interaction between FTIP1-like MCTPs and FT-like proteins are conserved in plants. Although GhMCTP3_A09 was from subfamily II, it had similar N-terminal C2 domain architecture to GhMCTP2_A08 and interacted with GhFT weakly (Additional file 1: Figure S1, Fig. 4b and 9). It would be interesting to examine whether AtFTIP3/4 (AtMCTP3/4), the orthologs of GhMCTP3_A09, interact with AtFT and GhMCTP3_A09 interact with AtSTM’s (AtFTIP3/4’s interacting protein) orthologs in cotton [7, 28]. GhMCTP16_A11, AtMCTP15 (QKY) and AtMCTP16 were from subfamily V, whose members were more divergent than members from subfamily I and II (Additional file 1: Figure S1) [7, 24, 27]. Therefore, further identification of the potential target proteins of various GhMCTPs is necessary to better understand GhMCTPs’ regulatory roles in cotton growth and development. Phylogenetic analysis of multiple C2 domains of 31 GhMCTPs showed that 4aC2, 4bC2 and 3aC2, 4cC2 and 3bC2, 4dC2 and 3cC2 were classified into subclade I, II, III, IV, respectively (Fig. 8), suggesting that different C2 domains of each GhMCTP might fulfill different functions. Whether these C2 domains bind specific membranes and interact with target proteins independently or cooperatively remains to be further studied.

## Conclusion

In our study, a systematic analysis of the multiple C2 domains and transmembrane region proteins (MCTPs) in *G. hirsutum* was performed to characterize their phylogenetic relationship, physicochemical properties, gene structures, domain architectures, conserved motifs and expression patterns. Furthermore, the N-terminus and the C-terminus of GhMCTPs were comparatively analyzed. GhMCTPs were classified into five subfamilies according to the phylogenetic tree. GhMCTPs within subfamiliy III, IV and V exhibited more diverse physicochemical properties, domain architectures and expression patterns than GhMCTPs within subfamily I and II. The distinct physicochemical properties between the N-terminus and the C-terminus suggested their distinct molecular functions in GhMCTPs. Yeast two-hybrid assay indicated that the N-terminus was responsible for GhMCTPs’ interaction with target proteins. Our study will benefit future studies on the functions of GhMCTPs in cotton growth and development.

## Methods

### Identification of GhMCTPs and their locations on chromosomes

The HAU.v1 version of genomic sequences and annotated gene models of *G. hirsutum* were downloaded from the CottonGen website (https://www.cottongen.org/data/download/genome_tetraploid/AD1) [43]. The protein sequence of AtFTIP1 (At5g06850) was used as the query to search against the protein database of *G. hirsutum* using BLAST with e-value threshold set at 1e-5 [25]. Then, all the BLAST hits were submitted to the SMART database (http://smart.embl-heidelberg.de/) to screen the putative GhMCTPs with 3–4 C2 domains in the N-terminus and 1–4 transmembrane regions in the C-terminus [44]. The full-length protein sequences of identified GhMCTP were aligned using Clustal Omega with default parameters (https://www.ebi.ac.uk/Tools/msa/clustalo/) [45]. The resulted alignment was used as the input file of MrBayes v3.2.5 to construct the phylogenetic tree with the evolutionary model set to the GTR substitution model with gamma-distributed rate variation across sites and Ngen, Samplefreq set to 300,000, 100, respectively [46].

The first amino acid to the right border of the last C2 domain and the remaining part in each GhMCTP were defined as the N-terminus and the C-terminus, respectively. The theoretical Mw, pI and GRAVY of the full length, N-terminus, C-terminus of GhMCTPs were calculated on the ExPASy website (http://web.expasy.org/protparam/) [47].

The chromosomal locations of *GhMCTPs* were obtained from the annotated gene models contained in the gff3 files and displayed by TBtools [48].

### Phylogenetic analysis of MCTPs in 27 plant species

MCTPs in 27 plant species (Additional file 8: Table S4) were identified with the same procedure used in the identification of GhMCTPs. The full-length protein sequences of all identified MCTP were aligned using Clustal Omega with default parameters (https://www.ebi.ac.uk/Tools/msa/clustalo/) [45]. The resulted alignment was submitted to the Gblock server to obtain the conserved sites in the alignment (http://molevol.cmima.csic.es/castresana/Gblocks_server.html), which were used as the input file of The MrBayes v3.2.5 to construct the phylogenetic tree with the evolutionary model set to the GTR substitution model with gamma-distributed rate variation across sites and Ngen, Samplefreq set to 2,000,000, 100, respectively [46]. The phylogenetic tree of 27 plant species was constructed using TBtools [48]. The whole genome duplication events that occurred during the evolution of these species were obtained from the publication of *Qiao* et al [36]. The MCTP numbers in these species and from different subfamilies were calculated using Excel 2013.

### Phylogenetic analysis of intron numbers of *MCTPs* in 26 plant species

The intron numbers of *MCTPs* in 26 plant species were obtained from the annotated gene models contained in the gff3 files. The intron numbers of *MCTPs* from different subfamilies were calculated using Excel 2013.

### Domain and conserved motif analysis

The lengths and positions of C2 domains and transmembrane regions in each GhMCTP were predicted by searching against the SMART database and displayed on the phylogenetic tree of GhMCTPs using iTOL v4 (https://itol.embl.de/) [49]. The conserved motifs in GhMCTPs were discovered using MEME v5.0.5 (http://meme-suite.org/tools/meme) with the following parameters: site distribution, zero or one occurrence per sequence; number of motifs, 6; motif width, between 6 and 50 [50].

### Synteny analysis and divergence time estimation

The MCScanX software (http://chibba.pgml.uga.edu/mcscan2/) was employed to detect syntenic *MCTPs* between A and D genome of *G. hirsutum*, *G. barbadense*, *G. raimondii, G. arboreum* according to the author’s manual [51]. These syntenic MCTPs were displayed using TBtools. The coding sequences of syntenic *MCTPs* were used to calculate Ka and Ks by TBtools with the NG method [48]. The divergence time was calculated according to the following formula: t = Ks/2r (r = 2.6 × 10^− 9^) [41].

### Expression analysis of *GhMCTPs* in different tissues

The transcriptome datasets of 23 cotton tissues were downloaded from the NCBI website under the BioProject PRJNA490626 (https://www.ncbi.nlm.nih.gov/bioproject/PRJNA490626), then transcriptomic reads were mapped against the *G. hirsutum* genome using HISAT2 and the read counts mapped on each gene were calculated using HTSeq v0.11.1 [52, 53]. Log2-transformed FPKMs of each *GhMCTP* in different cotton tissues were displayed on the heatmap using iTOL v4 (https://itol.embl.de/) [49].

### Phylogenetic analysis of C2 domains in the GhMCTPs

Four C2 domains of 4-C2-containing GhMCTPs and three C2 domains of 3-C2-containing GhMCTPs were designated as 4aC2, 4bC2, 4cC2, 4dC2 and 3aC2, 3bC2, 3cC2, respectively. The protein sequences of 107 C2 domains in the 31 GhMCTPs were extracted according to their lengths and positions in the full-length GhMCTPs. The obtained protein sequences were aligned using Clustal Omega with default parameters (https://www.ebi.ac.uk/Tools/msa/clustalo/) [45]. The MrBayes v3.2.5 was used to construct the phylogenetic tree with the evolutionary model set to the GTR substitution model with gamma-distributed rate variation across sites and Ngen, Samplefreq set to 300,000, 100, respectively [46].

### Yeast two-hybrid assay

The coding sequences of full length and N-terminus of *GhMCTP2_A08, GhMCTP3_A09* and *GhMCTP16_A11* were cloned into the pGADT7 vector (Clontech) and the coding sequence of *GhFT* (*Ghir_D08G024850.1*) [54, 55] was cloned into the pGBKT7 vector (Clontech) with the gene-specific primers (Additional file 9: Table S5). Then, different combinations of recombinant pGADT7 and pGBKT7 were co-transferred into the yeast strain Y2HGold which was cultured on DDO (SD/−Leu/−Trp) plates for 3 days. Three independent colonies on the DDO plates were chosen to test the interactions on QDO (SD/−Leu/−Trp/−His/−Ade) plates with 10 mM 3-AT (3-amino-1,2,4 -triazole).

## Supplementary information

**Additional file 1: Figure S1**. Phylogenetic tree of MCTPs in 26 plant species. A total of 368 identified MCTPs in 26 plant species are classified into five subfamilies and one outgroup according to the phylogenetic tree constructed by MrBayes v3.2.5. Both subfamily III and subfamily V are divided into a and b subclades. The probabilities that support the classified evolutionary subfamilies are marked on the branches of each partition in the tree. Stars and squares indicate MCTPs from *A. thaliana* and *G. hirsutum*, respectively. The tree scale bar represents 0.1 substitutions per amino acid.

**Additional file 2: Table S1**. Locations of C2 domains and transmembrane regions in GhMCTP proteins.

**Additional file 3: Figure S2**. Transmembrane helices in 31 GhMCTP proteins. TMHMM program (http://www.cbs.dtu.dk/services/TMHMM/) is used to predict the transmembrane helices. Red columns indicate the probabilities of the transmembrane helices, above which red blocks indicate the detected transmembrane helices.

**Additional file 4: Figure S3**. Syntenic *MCTPs* in *G. barbadense, G. raimondii and G. arboreum*. Blue and red bars represent chromosomes from A and D genome of *G. barbadense, G. raimondii* and *G. arboreum*, respectively. The grey lines link syntenic *MCTPs* detected by MCScanX. (A) Syntenic *MCTPs* between A and D genome of *G. barbadense*. (B) Syntenic *MCTPs* between D genome of *G. raimondii* and A genome of *G. arboreum*.

**Additional file 5: Table S2**. Coding sequence divergence between syntenic *MCTPs* in *G. barbadense*, G. raimondii and *G. arboreum*.

**Additional file 6: Table S3**. Physicochemical properties of the N-terminus and C-terminus of GhMCTP proteins.

**Additional file 7: Figure S4**. Multiple sequence alignment of GhMCTPs. the multiple sequence alignment is displayed in the interleaved format using Jalview v2.11. The darkness of the blue shades under the amino acids represent the identities: the darkest blue mark the highest identity. C2 domains and transmembrane regions are indicated below the alignment.

**Additional file 8: Table S4**. Information about 27 plant species used in identifying MCTPs.

**Additional file 9: Table S5**. Primer pairs used for gene clone in yeast two-hybrid assay.

## Data Availability

All data supporting the conclusions of this article are included in the article and its additional files. The HAU.v1 version of genomic sequences and annotated gene models of *G. hirsutum* can be downloaded from the CottonGen website (https://www.cottongen.org/data/download/genome_tetraploid/AD1). The websites where the genomic sequences and annotated gene models of all the 27 plant species can be downloaded are listed in the Additional file 8: Table S4. The transcriptome datasets of 23 cotton tissues can be downloaded from the NCBI website under the BioProject PRJNA490626 (https://www.ncbi.nlm.nih.gov/bioproject/PRJNA490626).

## Abbreviations

MCTP: Multiple C2 domains and transmembrane region protein
PD: Plasmodesma
ER: Endoplasmic reticulum
PM: Plasma membrane
SAM: Shoot apical meristem
GRAVY: Grand average of hydropathicity
Ka: Nonsynonymous substitution rate
Ks: Synonymous substitution rate
MYA: Million years ago
WGD: Whole-genome duplication
