# Supplementary figures and images for: Genome-wide identification and characterization of multiple C2 domains and transmembrane region proteins in Gossypium hirsutum
Source: BMC Genomics. 2020 Jun 29;21:445. doi: 10.1186/s12864-020-06842-1 (PMC7325108; doi:10.1186/s12864-020-06842-1)

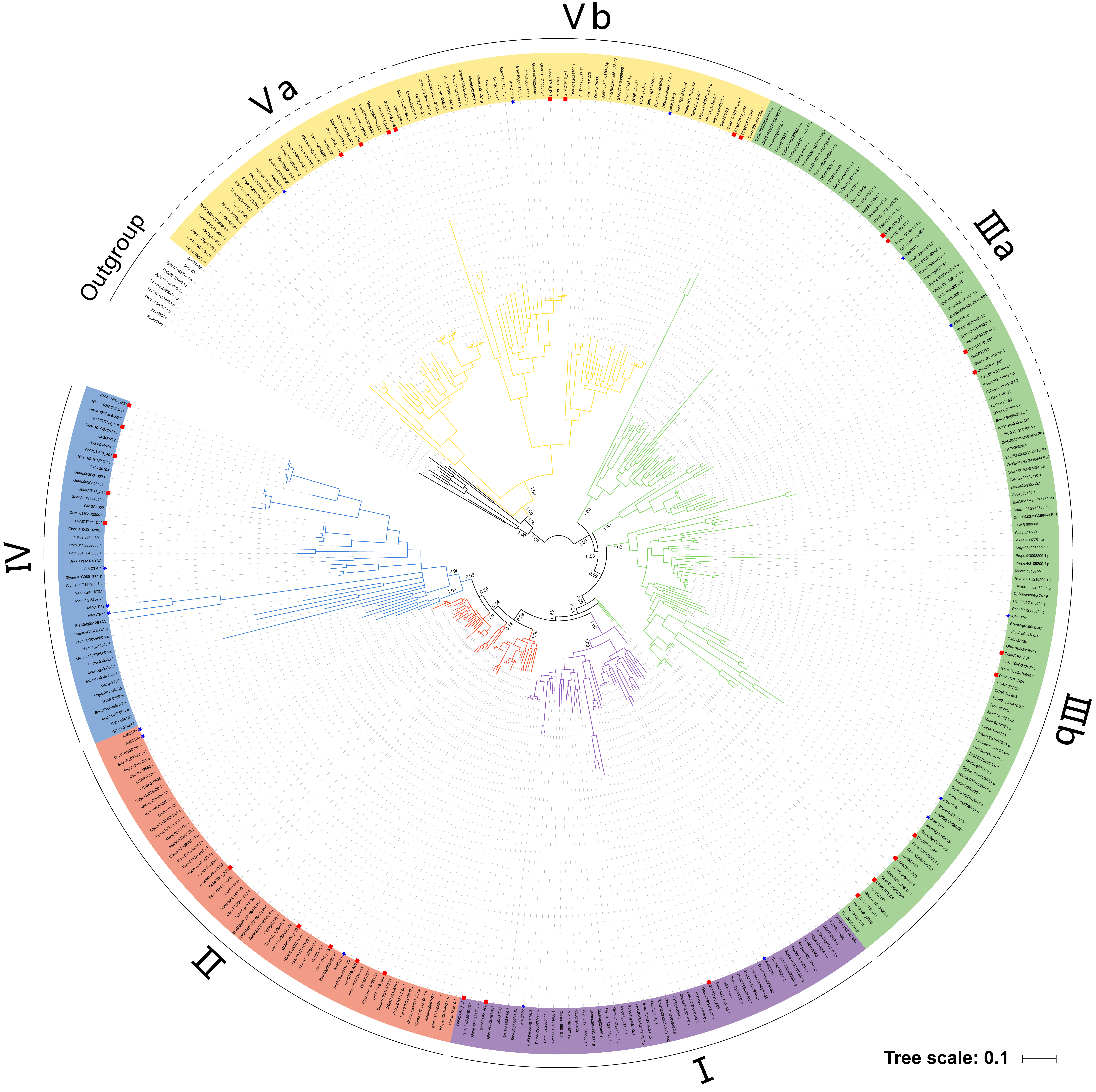

Supplement: Supplementary file 1 — Additional file 1: Figure S1. Phylogenetic tree of MCTPs in 26 plant species. A total of 368 identified MCTPs in 26 plant species are classified into five subfamilies and one outgroup according to the phylogenetic tree constructed by MrBayes v3.2.5. Both subfamily III and subfamily V are divided into a and b subclades. The probabilities that support the classified evolutionary subfamilies are marked on the branches of each partition in the tree. Stars and squares indicate MCTPs from A. thaliana and G. hirsutum, respectively. The tree scale bar represents 0.1 substitutions per amino acid. [file 12864_2020_6842_MOESM1_ESM.tif]

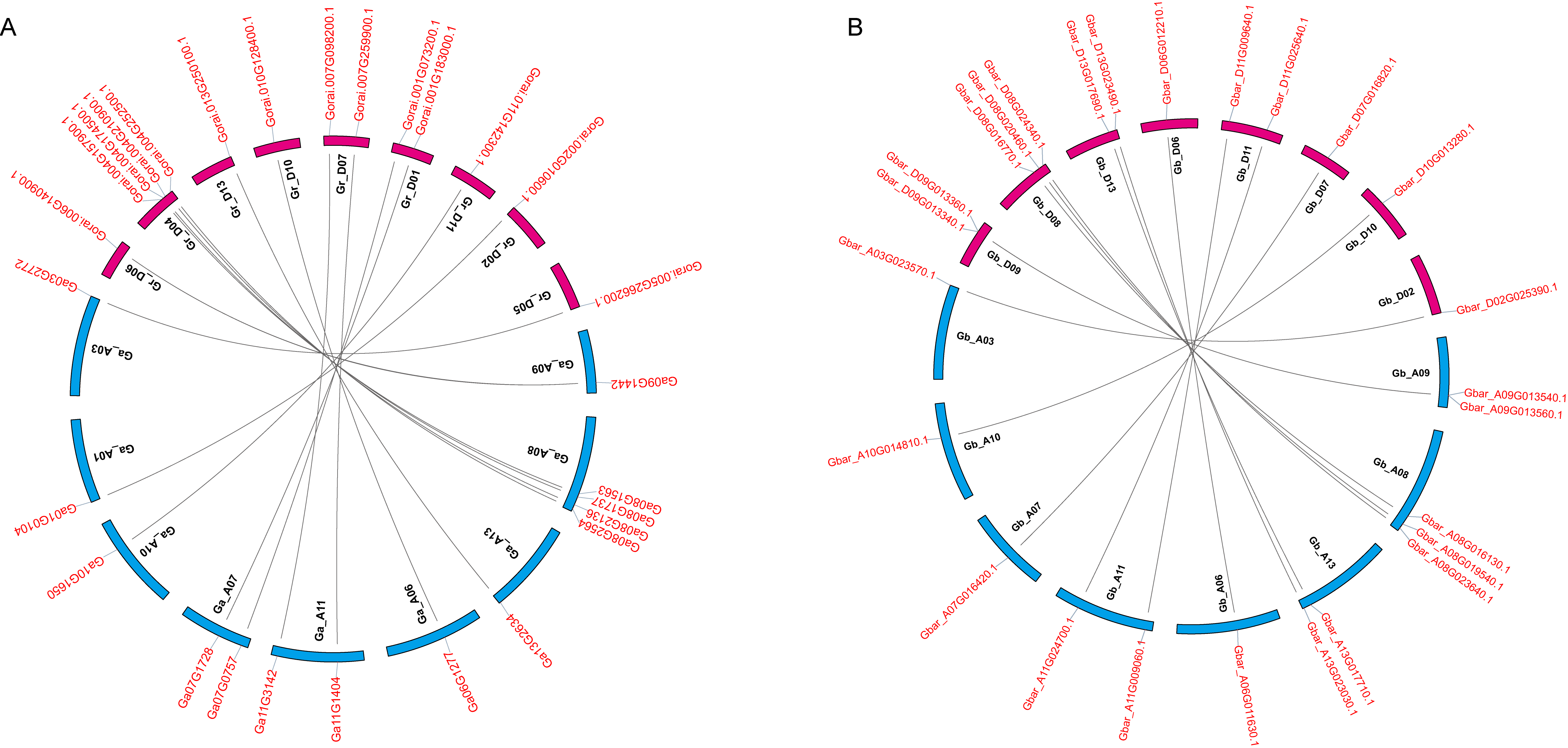

Supplement: Supplementary file 4 — Additional file 4: Figure S3. Syntenic MCTPs in G. barbadense, G. raimondii and G. arboreum. Blue and red bars represent chromosomes from A and D genome of G. barbadense, G. raimondii and G. arboreum, respectively. The grey lines link syntenic MCTPs detected by MCScanX. (A) Syntenic MCTPs between A and D genome of G. barbadense. (B) Syntenic MCTPs between D genome of G. raimondii and A genome of G. arboreum. [file 12864_2020_6842_MOESM4_ESM.tif]
